# The Probability of Inconstancy in Assessment of Cardiac Function Post-Myocardial Infarction in Mice

**DOI:** 10.4172/2329-6607.1000195

**Published:** 2016-09-09

**Authors:** Jiqiu Chen, Nadjib Hammoudi, Ludovic Benard, Delaine K Ceholski, Shihong Zhang, Djamel Lebeche, Roger J Hajjar

**Affiliations:** Cardiovascular Research Center, Icahn School of Medicine at Mount Sinai, New York, USA

**Keywords:** Myocardial infarction, Heart failure, Hemodynamics, Echocardiography, Statistics

## Abstract

In the present study, we explore the inherent variability that leads to overlaps in cardiac functional parameters between control and post-myocardial infarction (MI) mice. Heart failure was induced by Left Coronary Artery (LCA) ligation in mice. Average Ejection Fraction (EF) measured by echocardiography was lower in MI mice compared to control, but exhibited higher Standard Deviation (SD) and Standard Error (SEM), notably in 2D mode. Fractional Shortening (FS) showed a higher degree of overlap between MI and control mice even though the mean values were significantly different. Hemodynamic measurements of EF resulted in greater SD, SEM, ± 95% confidence intervals, and effect size. In comparing echocardiography at different time points, EF and FS were consistent by mean, but had apparent fluctuation in individual tracks, which were more obvious in MI than control mice. Hemodynamic measurements showed more complexity in data collection in mice *in vivo*. MI size showed variability that correlated with severity of cardiac function. These studies show that there is inherent variability in functional cardiac parameters after induction of heart failure by MI in mice. Analysis of these parameters by traditional statistical methods is insufficient, and we propose a more robust statistical analysis for proper data interpretation.

## Introduction

Left Ventricular (LV) Ejection Fraction (EF) and Myocardial Infarct (MI) size are the most important indicators of cardiac function in coronary ischemic heart failure [1,2]. Echocardiography and hemodynamic analyses are the most popular tools to detect heart function, and infarct size can be measured by echocardiography and direct digital photography [3,4]. Traditionally, heart failure is defined as an EF of less than 40% in human patients, but there is no standard definition for rodents [5,6]. Conventionally, if the EF post-MI is significantly lower than the control group, then the generation of a heart failure model is assumed [7]. Additionally, if heart function in MI mice is significantly improved after treatment, as determined by EF, it could be reported that the treatment is effective [8].

Statisticians have stressed that the P-value is not an absolute in indicating significance between two means [9,10]. The P-value is sensitive to multiple factors, including cohort number, where a high or low n can be manipulated into a low P value [11,12]. To date, there is no standard definition of heart failure in mice Changes in cardiac function are influenced not only by the size of the infarct but also by infarct location, anesthesia, echocardiographic probe, ventricular catheter position, animal’s body temperature, amount of bleeding, and operator experience [13,14]. Small animal surgery, echocardiography, and hemodynamic experiments are complex and prone to variability, as the small size of the mouse heart makes reproducibility difficult [14,15]. Most studies report the average EF or Fractional Shortening (FS) with Standard Deviation (SD) or Standard Error (SEM); however, the inherent variability of MI in mice isn’t exemplified in this practice. This, in addition to the lack of a standard definition of heart failure in mice, makes it difficult to conclude cardiac dysfunction and/or therapeutic improvement.

In the present study, we examine large cohorts of mice with and without MI to determine the level of variability in EF, FS, and MI size using 2D and M mode echocardiography and hemodynamic assessment. By reporting all animals examined, we show the range of EF and FS values that can be obtained and demonstrate the overlap between MI and control groups of mice. We also explore the correlation and margin of deviation between MI size and LV function. By reporting a more exhaustive set of statistical parameters (beyond mean +/− SEM and P-value), we demonstrate that a more powerful data set and comparisons can be generated. Overall, this study aims to provide a frame of reference for researchers to improve data collection and analysis of cardiac function in mice post-MI.

## Methods

### Animal protocol

All procedures were done according to the recommendations in the Guide for the Care and Use of Laboratory Animals (Department of Health and Human Services publication number NIH 78-23, 1996) and were approved by the Icahn School of Medicine at Mount Sinai Animal Care and Use Committee. C57BL6J male mice were used ranging in age from 10 to 12 weeks and in weight from 25–30 g. 70 mice underwent Left Coronary Artery (LCA) ligation to induce MI and 45 survived. 28 mice underwent sham operation for control and all survived. No animals were omitted from data analysis. Surgical procedures have been previously described [16]. Briefly, animals were anesthetized intraperitoneally with 0.06 mL mixture of KAX (ketamine 1 mL × 100 mg/mL, acepromazine 0.1 mL × 10 mg/mL, xylazine 0.1 mL × 20 mg/mL and 1 mL of 0.9% normal saline) per mouse. After thoracotomy, ligation of the LCA was performed with a 7-0 silk suture. The successful performance of LCA ligation was verified by visual inspection of the apex color. The chest was closed with 6-0 silk suture and the skin was closed with 4-0 silk sutures. Post-surgical monitoring was continued until the animal again becomes conscious. 8 mice died after LCA ligation (5 due to large MI area and bleeding, 3 because of additional anesthesia). 13 mice died 1–3 days after surgery due to heart failure. 4 mice were removed from the study because of signs of humane endpoints such as continual coma, cyanosis, and recumbence after 3 days. Antibiotics were not given, but no apparent infection developed in surviving animals during the course of the study or at the time of autopsy. All mice were housed under identical conditions and were given water and food *ad libitum*.

### Serial echocardiography

Echocardiograms were obtained at 1, 2 and 3 months after MI. Sham control mice were investigated at similar time points. The animals were sedated by intraperitoneal injection of ketamine (40 mg/kg). Echocardiograms were performed with GE Healthcare Echocardiography VIVID 7 system equipped with i13L-14 MHz probe. EF and FS data were obtained using M mode and 2D mode echocardiography following the vendor’s User Manual as previously described [17,18]. From LV parasternal 2D long-axis view, LV end-diastolic and LV end-systolic volumes were derived from the area-length method using a prolate-ellipsoid formula (volume=8*[A^2^/(L*3*π)], A=LV area, L=LV length, Simpson’s method). LV Stroke Volume (SV) was calculated as the difference between the two LV volumes and LVEF was calculated (LVEF=[(LVSV)/LV end-diastolic volume]*100) [19]. MI size (%) was acquired by measuring LV apical infarct length divided by U-type LV length. LV mass was derived by cubic method (LVmass=(LV internal diameter, diastolic (LVIDd) + posterior wall (PW) + anterior wall (AW))^3^ − (LVIDd)^3^) [20].

### *In vivo* hemodynamics

Pressure-Volume (PV) loops of the LV were acquired and analyzed as previously described [14,21]. Briefly, hemodynamic measurements were performed using a 1.2 Fr PV conductance catheter (Scisense). Mice were injected intraperitoneally with urethane (1 g/kg), etomidate (10 mg/kg) and morphine (1 mg/kg) before inhalation of 5% (vol) isoflurane, then mechanically ventilated and maintained at 0.5–1% (vol) isoflurane during the surgical procedure. The right carotid artery was exposed and isolated via a midline incision. Three sutures (7-0 silk) were placed under the left common carotid artery. The top one was tied and the bottom one was pulled toward to heart to prevent bleeding. A small incision was made in the middle of the carotid artery then the catheter was introduced and advanced down the ascending aorta, through the aortic valve, and into the LV. A small incision up the diaphragm was made and followed by transient (1–2 s) occlusion of the thoracic vena cava to decrease venous return during the recording of hemodynamics. The left jugular vein was intubated with a 24 G catheter connected to a 1 cc syringe with ~0.8 mL saline and 0.1% heparin. Subsequently, parallel conductance was determined by a 50 μL injection of 0.9% saline into the jugular vein. 35 MI mice underwent PV loops and 8 MI mice were excluded due to technical issues, like bleeding or bad catheter positioning. 15 control mice underwent PV loops and all were included.

### MI by direct imaging

Hearts were harvested after the final hemodynamic measurement. The hearts were perfused with 10 mL cold PBS and 1% heparin. The LV was cut from the root of the pulmonary artery to the ventricular apex and photographed. MI size (% area) was the average of the infarct area of the whole heart, left half of the LV, and right half of the LV and quantified using Image-Pro software (Media Cybernetics, Bethesda, MD, USA) [21].

### Statistics

Variables are expressed as mean ± Standard Error of the Mean (SEM). One-way ANOVA was used for time course of heart function and Student’s *t*-test was used for all other statistical analyses to compare experimental groups using GraphPad Prism software. P-values <0.05 were considered statistically significant. Cohen’s effect size based on the difference between two means was calculated by dividing the difference between means by the standard deviation (0.2–0.3 is considered a small effect and 0.8 or higher is considered a large effect).

## Results

### Mean values and overlapping of LV functions in M mode and 2D mode echocardiography post-MI

**Figure 1:**
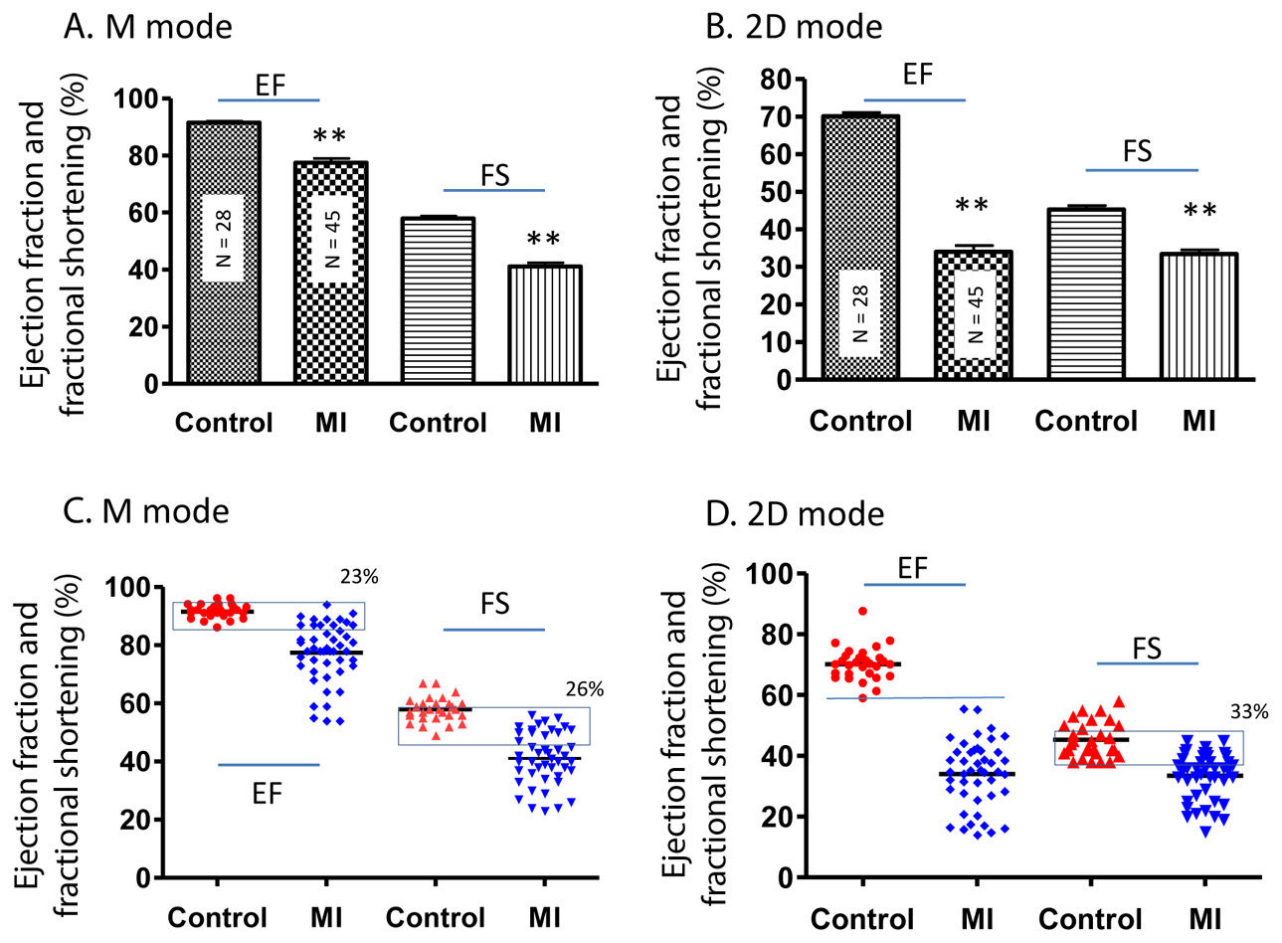
Mean value and scatter plot of LVEF and FS in M and 2D mode echocardiography in mice. EF and FS as determined by M mode (A, C) and 2D mode (B, D) echocardiography presented as the mean ± SEM (A, B) or scatter plot with the mean shown as a black line (C, D). ^**^P<0.01 compared with control. In (C) and (D), the % overlap between control and MI groups is provided and overlapping samples are shown in a box.

In general, cardiac function was significantly worse in MI compared to control mice. In the MI group, the LV anterior wall thickness was decreased, LV internal diastolic and systolic diameter were increased (Table 1). The mean values of EF and FS were significantly lower in the MI group compared to controls (P<0.01) (Figures 1A and 1B). However, scatter plot analysis of this data revealed that 23% and 26% of MI mice had overlapping EF and FS with control mice, respectively (Figure 1C). The EF determined by M mode was much higher compared to 2D mode for both MI and control mice (Figure 1D). As evidenced by the scatter plots, 2D mode resulted in more variation in EF and FS compared to M mode both in MI and control groups even though the standard error is very low due to high animal number.

### Time course variation of heart function with 2D mode

**Figure 2:**
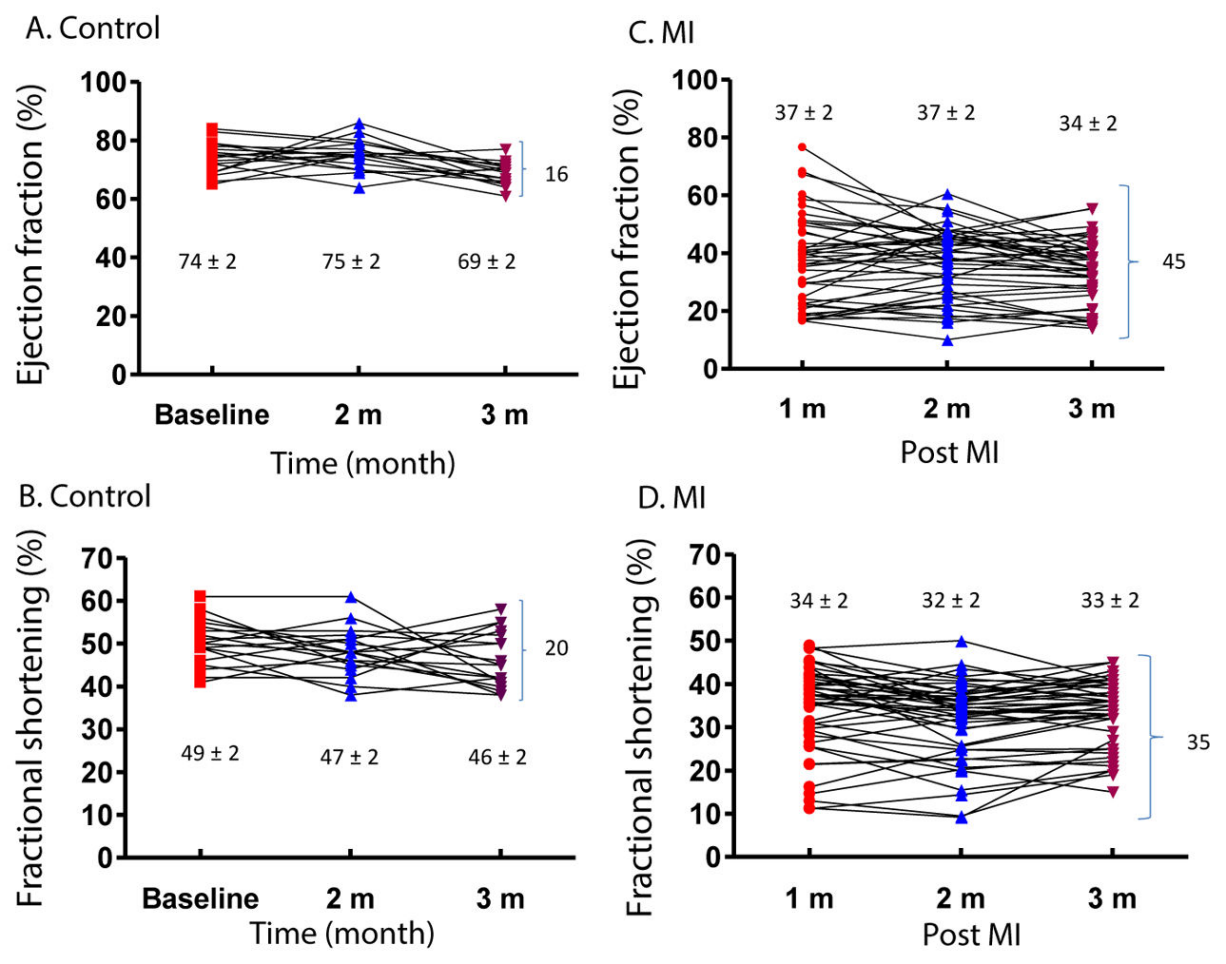
Time course of 2D mode echocardiography on heart function in mice. EF (A) and FS (B) are shown for each individual animal in the control group over a 3 month period (n=18). EF (C) and FS (D) are shown for each individual animal in the MI group over 3 months (n=45). The mean ± SEM (%) is shown at each time point and P>0.05 when comparing each time point in both control and MI groups. Variance (%) in EF and FS between individual animals is also shown in braces for each group. MI mice showed more variation in EF and FS between time points; m=month.

2D mode echocardiography was performed on control and MI mice at different time points. 39% (7/18) of control and 29% (13/45) of MI mice had fluctuations of 5–10% in EF, and 17% (3/18) of control and 22% (10/45) of MI mice had fluctuations >10% in EF in a one-month interval in time course measurements. There were similar discrepancies in FS values for both control and MI groups (Figure 2). Analysis of EF and FS in individual mice over time demonstrates the range of values that can be obtained, notwithstanding a virtually identical average between time points with a P-value >0.05. For EF, the difference between the highest and lowest values was 16% in control and 45% in MI mice (Figures 2A and 2C). For FS, the difference was 20% and 35% in control and MI mice, respectively (Figures 2B and 2D). This occurred despite the fact that the standard error of the mean was very small (<2%) (Figure 1B).

### Variation of cardiac function associated with MI size

**Figure 3:**
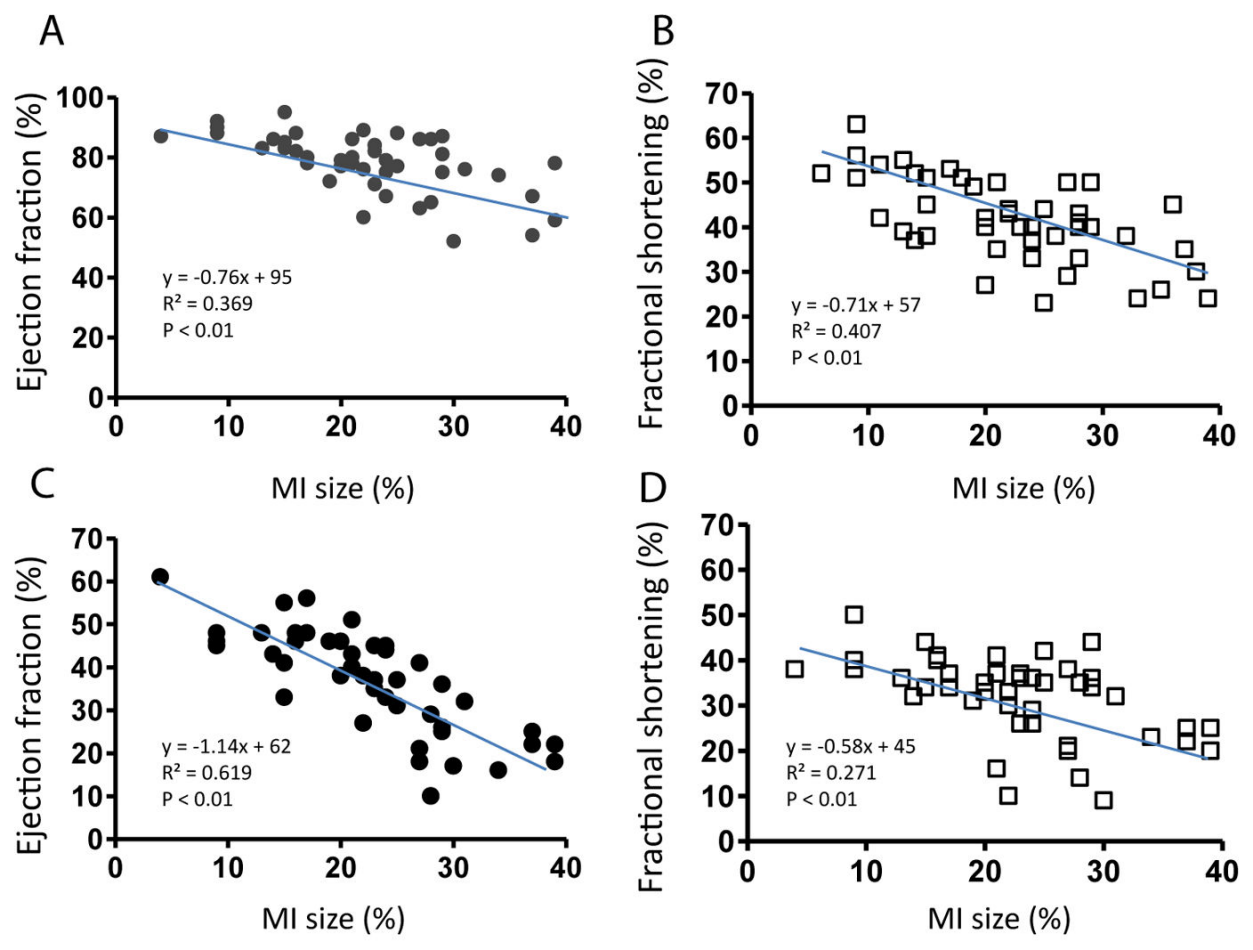
Correlation between MI size (determined by echocardiography) and EF or FS by M mode (A and B) or 2D mode (C and D) echocardiography in MI mice. The equation of the line, correlation coefficient (R^2^), and P-value of the trend are shown.

**Figure 4:**
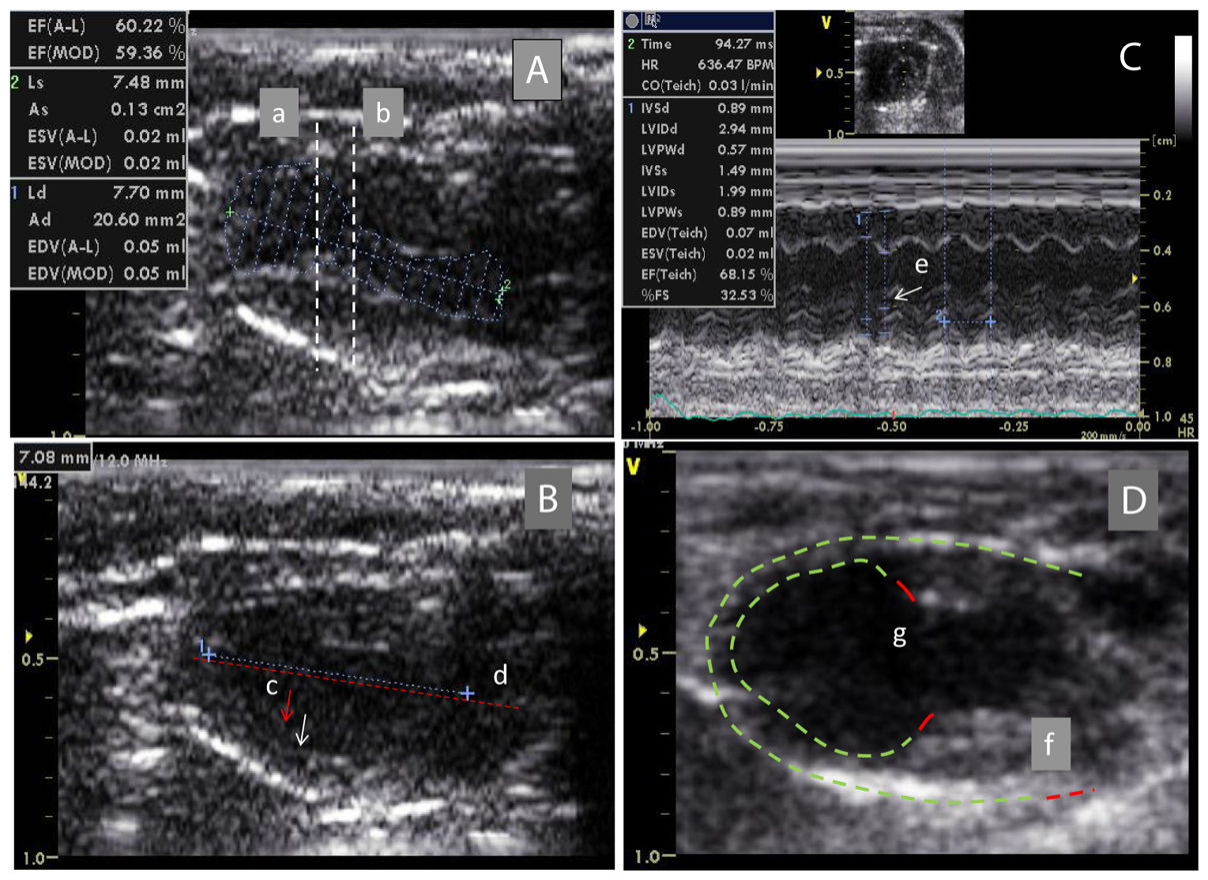
Potential causes of variability of functional and morphological assessment. (A) 2D mode of systolic LV with a MI located at apex. Probe at (a), EF=79%; probe at (b), EF=92%. (B) Diastolic LV echo shows that the internal edge (c; arrows) and end of outflow tract (d; dashed lines) were not clear in some hearts. (C) In M mode echocardiography, the choice of posterior border (g) was difficult in some cases due to hypertrophic papillary muscle. (D) MI area measurement. There is no standard to choose the starting and end point of LV (f) and MI length (g). The boundary between the infarct and normal zone could be identified anywhere along a segment (red line) *in vivo*, leading to measurement variability.

**Figure 5:**
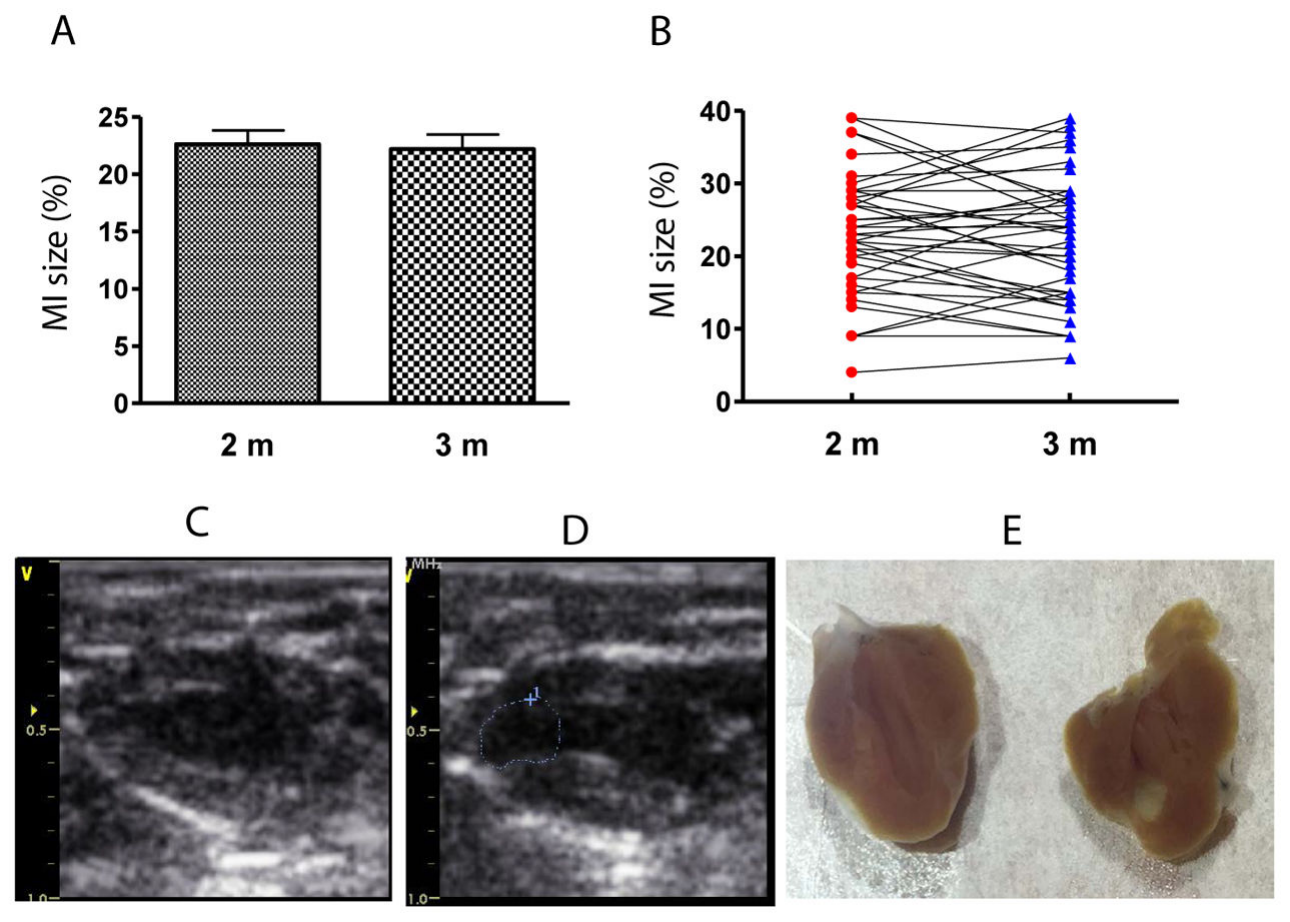
Variation in MI size as measured by echocardiography. (A) Average MI size showed no significant difference between 2 and 3 months (2 m and 3 m, respectively) post-surgery. (B) MI size shown as a scatter plot showed apparent discrepancy between animals. (C) Echocardiography at 2 month time point showed almost no evidence of MI. (D) Echocardiography from the same animal as shown in (C) at 3 month time point showed moderate MI (blue dotted circle). This difference could be due to the ultrasonic probe being moved slightly leftward from the midline (<0.5 mm). (E) Direct imaging of heart cut longitudinally to confirm MI in the mouse shown in (C) and (D), where the MI is mainly located on the left side of the LV.

MI size was determined by echocardiography. Figure 3 shows that EF and FS negatively correlated with MI size (P<0.01). However, there were variations greater than 20% in EF and FS using either M or 2D mode at most MI size points. Figure 4 shows some factors that typically cause this variation. The echo probe level has a significant impact on M mode data; a 1 mm difference in placement can result in 15–20% variations in EF (Figure 4A). Another cause for variation in 2D mode echocardiographic measurement of EF can be due to an unclear internal edge of the LV and end of outflow tract (aortic valve) (Figure 4B). In M mode echocardiography, the choice of posterior border can be difficult in some cases due to hypertrophic papillary muscle (Figure 4C). Regarding MI size, the determination of the left and right points of U-type LV length and the border point of infarction is complicated, as the infarct wall shows gradients in thickness (Figure 4D). Figure 5A shows that the average MI size 2–3 months after surgery is consistent by echocardiography, but scatter plot reveals that MI size fluctuated by 5–10% in 36% of mice between the 2- and 3-month time points, while 10% of mice showed >10% fluctuation in MI size in this same period (Figure 5B). One of the common causes of inconsistency in MI size is the angle of the echocardiographic probe in relation to the LV long axis. Small or moderate MIs induced by occlusion of the small branch of the LCA could potentially be overlooked if the transducer probe was placed in the standard view position on the long axis because the infarct occurred on the left side of the LV (Figures 5C–5E).

### Variation in hemodynamics and digital photo of MI

**Figure 6:**
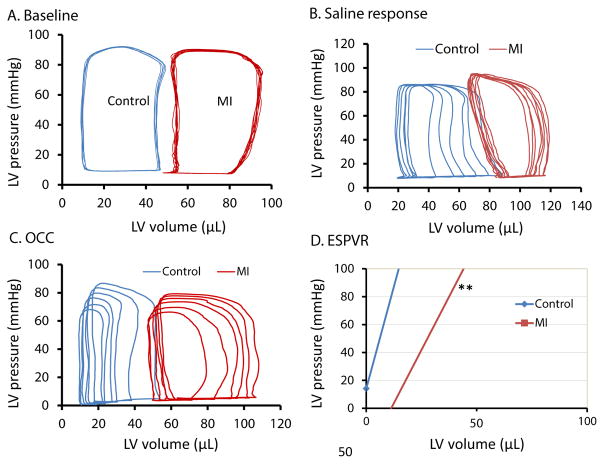
Cardiac function measured by hemodynamics in control and MI mice. (A) Baseline of PV loops. Note that the PV loops were not very regular in most MI mice due to uneven LV geometry, which could result in a large variation in EF data in MI mice. (B) PV loops after injection of 50 μL of 0.9% saline. EF significantly increased with EDV in controls, but this drastic change was not recapitulated in MI mice. (C) Occlusion (OCC; 1–2 s) of the thoracic vena cava to decrease venous return. The variation after OCC is larger and it’s difficult to obtain a similar result in two different assays in the same animal. (D) End-Systolic Pressure Volume Relationship (ESPVR) between LV pressure and LV volume. Animal number: control=15, MI=27, P<0.01 compared with control.

**Figure 7:**
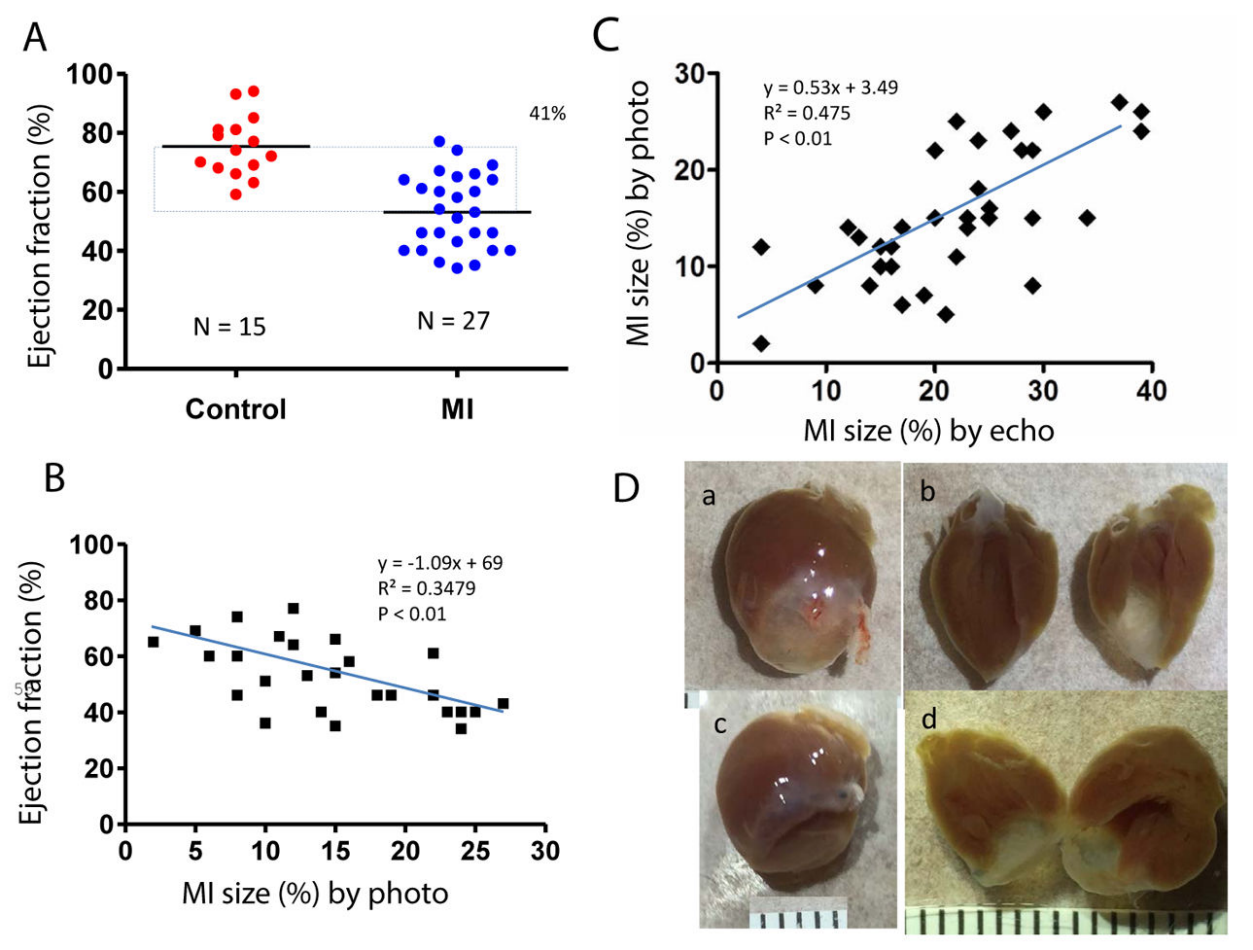
Hemodynamics and MI. (A) Average EF (mean ± SEM) is significantly lower in MI mice compared to controls (^**^P<0.01). Scatter plot data shows that 41% EF values overlapped between the two groups. (B) EF is negatively correlated with MI size measured by direct imaging of the heart. (C) Correlation of MI size measured by direct imaging and echocardiography. (D) Direct imaging of MI: (a) Whole heart, (b) Opened LV, (c) LV aneurysm with ventricular wall depressed, and (d) LV wall was curled, affecting measurement of MI size.

**Figure 8:**
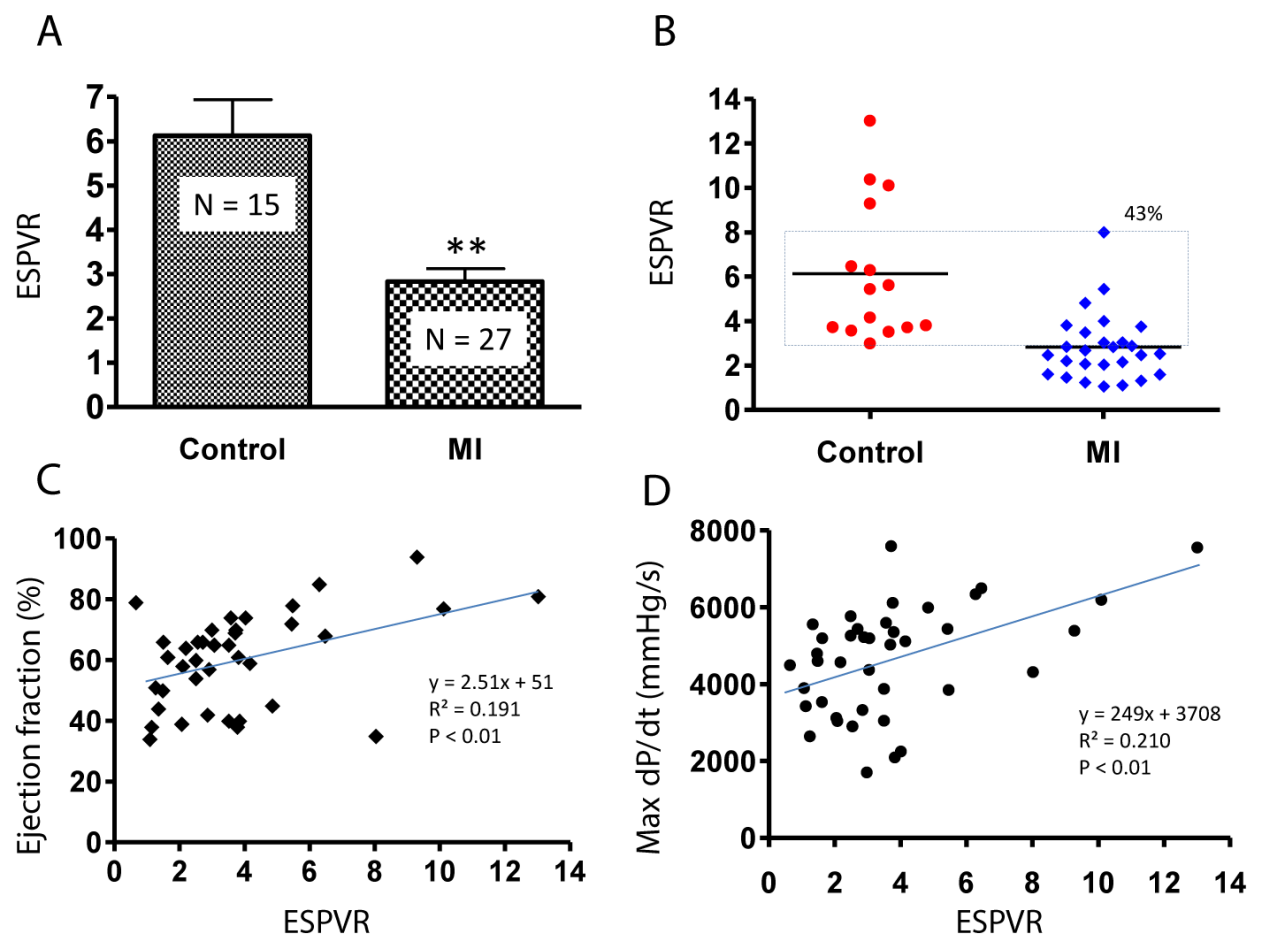
Correlation between End-Systolic Pressure Volume Relationship (ESPVR) and EF or max dP/dt. (A) Average ESPVR (mean ± SEM) in control and MI groups (^**^P<0.01 compared to control). (B) Data shown as a scatter plot reveals a 43% overlap of ESPVR in MI and control groups. Correlation between ESPVR and EF (C) or max dP/dt are shown. The equation of the line, correlation coefficient (R^2^) and P-value of the trend line are shown.

PV loop data showed significant differences in ± dP/dt, Tau, ventricle volume, stroke volume, and LV weight in the MI group compared to controls (Table 2). Figure 6 shows PV loop examples of steady-state, saline-treated, transient occlusion (OCC) of the thoracic vena cava, and end-systolic PV relationships (ESPVR) in control and MI mice. The average EF was significantly decreased in MI compared to control mice (P<0.01), but scatter plot analysis of this data revealed that 41% of EF values overlapped between the MI and control groups (Figures 7A and 7B). EF negatively correlated with MI size measured by direct imaging, even though the EF varied by 30–40% for most MI size points. Positive correlation was found in MI size measured by echocardiography and direct imaging. However, there was apparent discrepancy in MI size by these two methods, as the R^2^ value was only 0.475 (Figure 7C). As shown in Figure 7D, the heart is oval in shape; therefore, it is difficult to measure MI size as a percentage of heart surface area by a regular digital photo. When the heart is opened to assess MI size, curling of the infarcted wall, due to wall thinning as a direct consequence of MI, could result in a smaller MI size (Figure 7D; a and b). ESPVR values had similar variability to EF in both the control and MI groups (Figures 8A and 8B) even though the ESPVR positively correlated with EF and max dP/dt (P<0.01) (Figures 8C and 8D).

## Discussion

In this study, we examined EF, FS, and MI size in a large cohort of mice post-MI by echocardiography and hemodynamics in order to determine the range of values typically observed in these methods. In addition to reporting the mean, standard error, and P-value, we analyzed the data by multiple statistical methods in order to gage a truthful comparison between functional measurements. The inherent variability in induction of MI in small rodents results in high variability in functional parameters. The data from the 70 mice presented in this study can be used as a reference index for subsequent heart failure studies in small rodents, as our large sample size and robust statistical analysis could provide a frame of reference for a typical data set.

In the examination and assessment of heart failure models in mice, several parameters are obtained, such as EF, FS, end-diastolic volume, and end-systolic volume [22,23]. A significant difference in these parameters, as evaluated by traditional hypothesis testing, provides evidence of defective cardiac function. In our study, the averages of all key functional parameters were significantly lower in MI than control mice; with a P-value <0.01, this signifies that the probability that a specific parameter of a control mouse is identical to that of a MI mouse is less than 1%. Therefore, if only the average is considered, one could conclude that, based on the data presented in this paper, a successful MI model of heart failure was established using LCA ligation. However, there is inherent variability between mice, as is evidenced by the presentation of our data as scatter plots. For example, if a mouse has a FS of 52% as obtained by M mode echocardiography, it would be impossible to determine if this mouse has heart failure, as 15% of MI mice had FS ≥ 52% while 17% of control mice had FS ≤ 52%. We could not be sure that the change in EF is due to experiment injury or medical treatment (Figure 2).

In the present study, we examine the variability that occurs in assessment of MI by echocardiography and PV loop. Cardiac function data in mice post-MI are variable between laboratories [24,25]; in some instances, the EF can be as low as 21% with a standard error of less than 1% even in a group of 5–7 mice [8,26]. Our data show that EF values measured by M mode are higher than 2D mode echocardiography, but there is a higher chance of obtaining P values <0.01 due to low deviation and small scatter range. FS values measured in M mode have lower overall values but a higher overlap rate and SD/mean ratio. Manual analysis of LV volume can be adapted to different pathophysiology, but is usually accompanied by a higher deviation (Figures 2C and 4B). 2D mode echocardiography had the lowest percentage overlap rate and a higher effect size between the MI and control groups (Table 3). However, it also produced a larger range of values (42%) and a higher SD/mean ratio (32%) in EF in MI mice; thus, the probability of data inconstancy is higher in 2D mode than M mode, particularly with small sample sizes even if the P-value <0.01. PV loops are an invasive process and it is not possible to repeat them in the same animal. The geometry of the heart changes post-MI and the aneurysm makes the loops irregular, resulting in variation of EF. Compared to echocardiography, EF determined by PV loops had a higher effect size, SD/mean ratio, 95% confidence intervals, range of scatter, and percentage overlap rate (Table 3). This means that if the animal number is small, it will be difficult to draw conclusions from this type of data [11].

In summary, we have characterized a large cohort of control and MI mice using multiple methods to evaluate functional parameters. We have provided a robust statistical analysis of EF and FS in 2D and M mode echocardiography and hemodynamic analysis by PV loops. We suggest that researchers use more than one method to determine EF and FS, and if echocardiography is used, the mode should be clearly stated (as evidenced by the different values obtained with 2D and M mode). The data should also be analyzed beyond statistic difference, and authors should report 95% confidence intervals and overlap rate percentage between experimental groups, as these parameters better demonstrate the variability of the data compared to P-value alone. We also recommend that authors publish how many animals were excluded from the study (and reasons why) and how many animals died during the procedures, as these are not always provided. Our study has two limitations: 1) The variability in functional parameters following a treatment post-MI was not examined; and 2) We did not pursue any technical reasons for variability in MI size in mice. We are currently addressing this latter limitation by exploring the diversity of LCA in relation to MI size in mice.

## Figures and Tables

**Table 1:** Left ventricle function as determined by echocardiography in mice post-MI.

	No.	IVSd (mm)	LVIDd (mm)	LVPWd (mm)	IVSs (mm)	LVIDs (mm)	LVPWs (mm)	EDV (μL)	ESV (Teich) (μL)	HR (BPM)	LV mass (mg)	BW (g)
Control 1 m	18	0.94 ± 0.02	2.61 ± 0.06	0.84 ± 0.01	1.58 ± 0.06	1.01 ± 0.05	1.47 ± 0.04	50 ± 4	1.0 ± 0.1	534 ± 8	92 ± 3	28 ± 0.5
Control 2 m	18	0.97 ± 0.03	2.63 ± 0.09	0.89 ± 0.05	1.63 ± 0.03	1.02 ± 0.05	1.48 ± 0.03	51 ± 5	1.1 ± 0.1	520 ± 11	90 ± 3	29 ± 0.7
Control 3 m	28	1.04 ± 0.02	2.62 ± 0.07	0.96 ± 0.03	1.64 ± 0.03	1.10 ± 0.04	1.48 ± 0.04	50 ± 4	3.6 ± 0.1	549 ± 5	92 ± 2	31 ± 0.4
MI 1 m	45	0.71 ± 0.04**	3.18 ± 0.09**	1.00 ± 0.06	1.08 ± 0.17**	1.89 ± 0.07**	1.28 ± 0.06**	85 ± 6**	20 ± 5.1**	510 ± 7	102 ± 3**	27 ± 0.3
MI 2 m	45	0.68 ± 0.02**	3.13 ± 0.06**	0.89 ± 0.02	0.99 ± 0.03*	1.83 ± 0.07**	1.36 ± 0.03*	84 ± 5**	20 ± 2.4**	479 ± 6	110 ± 4**	29 ± 0.3
MI 3 m	45	0.66 ± 0.03**	3.19 ± 0.07**	0.91 ± 0.03	1.03 ± 0.03**	1.88 ± 0.08**	1.39 ± 0.04	87 ± 6**	23 ± 3.1**	456 ± 6	114 ± 4**	30 ± 0.3

No: Animal Number; IVSd: Inter-Ventricle Septum, Diastolic; IVSs: Inter-Ventricle Septum, Systolic; LVIDd: LV Internal Diameter, Diastolic; LVIDs: LV Internal Diameter, Systolic; LVPWd: LV Posterior Wall, Diastolic; LVPWs: LV Posterior Wall, Systolic; EDV: End-Diastolic Volume; ESV: End-Systolic Volume; LV: Left Ventricle; HR: Heart Rate; BPM: Beats Per Min; BW: Body Weight; m: Month.

* P<0.05;

** P<0.01 compared with control.

**Table 2:** Hemodynamic measurement of left ventricle function in mice post-MI.

	No.	Max P (mmHg)	Min P (mmHg)	Max dP/dt (mmHg/s)	Min dP/dt (mmHg/s)	Tau (ms)	EDV (μL)	ESV (μL)	SV (μL)	CO (mL/min)	SW (μL* mmHg)	HR (BPM)	BW (g)	LVW (mg)
**Control**	15	80 ± 3	7 ± 0.6	5324 ± 396	−4253 ± 332	8 ± 0.6	51 ± 3	19 ± 2	40 ± 2	18 ± 1	2664 ± 180	454 ± 23	31 ± 0.6	122 ± 3
**MI**	27	85 ± 2	8 ± 0.5	4293 ± 235*	−3100 ± 195**	11 ± 0.5**	82 ± 5**	50 ± 4**	46 ± 2*	16 ± 0.6	2727 ± 126	349 ± 9**	29 ± 0.3	148 ± 3**

Max P: Maximum Pressure; Min P: Minimum Pressure; EDV: End-Diastolic Volume; ESV: End-Systolic Volume; SV: Stroke Volume; CO: Cardiac Output; SW: Stroke Work; HR: Heart Rate; BPM: Beats Per Min; BW: Body Weight; LVW: Left Ventricle Weight.

* P<0.05;

** P<0.01 compared with control.

**Table 3:** Variance in EF and FS as measured by echocardiography and hemodynamics.

	M mode				2D mode				PV loops	
	EF		FS		EF		FS		EF	
	Control	MI	Control	MI	Control	MI	Control	MI	Control	MI
**Mean (%)**	92	77**	58	42**	70	34**	45	33**	75	53**
**N; number of mice**	28	45	28	45	28	45	28	45	15	27
**SD (%)**	2.3	10.2	4.3	9.4	5.5	10.9	5.8	7.5	10.2	12.4
**SD/Mean ratio**	2.5	13.2	7.4	22.4	7.9	32.1	12.8	22.7	13.6	23.4
**SEM (%)**	0.44	1.49	0.81	1.37	1.02	1.62	1.07	1.12	2.65	2.38
**+95% CI (%)**	93	80	60	44	72	37	47	36	81	68
**−95% CI (%)**	91	75	56	39	68	31	43	31	70	38
**Highest value (%)**	96	94	67	63	88	56	58	45	94	77
**Lowest value (%)**	86	54	49	23	59	14	38	15	59	34
**Range of scatter (%)**	10	40	18	40	29	42	20	30	35	43
**Overlap rate (%)**	-	23	-	26	-	0	-	33	-	41
**Effect size**	-	0.721	-	0.754	-	0.967	-	0.775	-	0.97

EF: Ejection Fraction; FS: Fractional Shortening; MI: Myocardial Infarction; No: Animal Number; SD: Standard Deviation; SEM: Standard Error of Mean; CI: Confidence Interval; Overlap rate: (Overlapped animal number/Total animal number) × 100.

** P<0.01 compared with control.

## Abbreviations

MI: Myocardial Infarction
LV: Left Ventricle
LCA: Left Coronary Artery
EF: Ejection Fraction
FS: Fractional Shortening
PV: Pressure-Volume
SD: Standard Deviation
SEM: Standard Error of Mean
